# Ultrasound-guided percutaneous peripheral nerve stimulation for analgesia following total knee arthroplasty: a prospective feasibility study

**DOI:** 10.1186/s13018-016-0506-7

**Published:** 2017-01-13

**Authors:** Brian M. Ilfeld, Christopher A. Gilmore, Stuart A. Grant, Michael P. Bolognesi, Daniel J. Del Gaizo, Amorn Wongsarnpigoon, Joseph W. Boggs

**Affiliations:** 1 Department of Anesthesiology, University of California San Diego, 200 West Arbor Drive, MC 8770, San Diego, CA 92103-8770 USA; 2 Department of Anesthesiology, Wake Forest University Baptist Medical Center, 145 Kimel Park Drive, Ste 330, Winston-Salem, NC 27103 USA; 3 The Center for Clinical Research, Winston-Salem, NC USA; 4 Carolinas Pain Institute, Winston-Salem, NC USA; 5 Department of Anesthesiology, Duke University Medical Center, DUMC 3094, Durham, NC 27710 USA; 6 Department of Orthopedic Surgery, Duke University Medical Center, 200 Trent Dr. #5216, Durham, NC 27710 USA; 7 Department of Orthopedic Surgery, University of North Carolina, 3147 Bioinformatics Building, 130 Mason Farm Road, Chapel Hill, NC 27599-7055 USA; 8 SPR Therapeutics, LLC, 22901 Millcreek Blvd, Suite 110, Cleveland, OH 44122 USA

**Keywords:** Ultrasound-guided analgesia, Peripheral nerve stimulation, Postoperative analgesia, Acute pain, Neuromodulation, Neurostimulation, Ultrasound-guided percutaneous peripheral nerve stimulation, Orthopedic surgery, Total knee arthroplasty, Total knee replacement

## Abstract

**Background:**

Peripheral nerve stimulation has been used for decades to treat chronic pain but has not been used for postoperative analgesia due to multiple limitations, beginning with invasive electrode placement. With the development of small-diameter/gauge leads enabling percutaneous insertion, ultrasound guidance for accurate introduction, and stimulators small enough to be adhered to the skin, neurostimulation may now be provided in a similar manner to continuous peripheral nerve blocks. Here, we report on the use of ultrasound-guided percutaneous peripheral nerve stimulation to treat postoperative pain.

**Materials and methods:**

Subjects within 60 days of a total knee arthroplasty with pain insufficiently treated with oral analgesics had a 0.2-mm-diameter electrical lead (pre-loaded into a 20 gauge needle) introduced percutaneously using ultrasound guidance with the tip located approximately 0.5–1.0 cm from the femoral nerve (a second lead was inserted approximately 1.0–3.0 cm from the sciatic nerve for posterior knee pain). An external stimulator delivered current. Endpoints were assessed before and after lead insertion and the leads subsequently removed. Due to the small sample size for this pilot/feasibility study, no statistics were applied to the data.

**Results:**

Leads were inserted in subjects (*n* = 5) 8–58 days postoperatively. Percutaneous peripheral nerve stimulation decreased pain an average of 93% at rest (from a mean of 5.0 to 0.2 on a 0–10 numeric rating scale), with 4 of 5 subjects experiencing complete resolution of pain. During passive and active knee motion pain decreased an average of 27 and 30%, respectively. Neither maximum passive nor active knee range-of-motion was consistently affected.

**Conclusions:**

Ultrasound-guided percutaneous peripheral nerve stimulation may be a practical modality for the treatment of postoperative pain following orthopedic surgical procedures, and further investigation appears warranted.

## Background

Total knee arthroplasty often results in moderate-to-severe pain that is frequently treated with opioids, themselves correlated with unwelcome side effects such as sedation, nausea, vomiting, respiratory depression, and abuse [1]. Other analgesic techniques such as continuous peripheral nerve blocks have their own limitations as they induce quadriceps weakness and are associated with an increased risk of falling [2]; duration limitations because of the risk of infection [3]; and, for outpatients, the encumbrance of carrying the local anesthetic reservoir and portable infusion pump [4]. An alternative analgesic modality—peripheral nerve stimulation—may deliver post-surgical pain control without the limitations of currently available analgesic techniques.

Peripheral nerve stimulation was initially described to treat pain over 2,000 years ago with the use of the electrical charge generated by Torpedo Fish [5]. Many hypotheses have been proposed to explain the analgesic effects of stimulation [6], but Melzack and Wall’s “gate control theory” is the most common and accepted theory [7]. In 1967, Melzack and Wall described how large-diameter myelinated afferent peripheral nerve fibers were activated by electrical current which, in turn, impeded pain signal conduction (the “gate”) within the spinal cord, to the central nervous system from small-diameter pain fibers [7, 8]. Soon thereafter, Wall and Sweet hypothesized that stimulating primary afferent neurons could produce analgesia [9]. Subsequently, off-label use of commercially available stimulators was described to deliver nerve stimulation to peripheral nerves [10]. In the past 40 years, surgically implanted peripheral nerve and spinal cord stimulators have been validated and thoroughly investigated in managing *chronic* pain [11, 12].

However, using neurostimulation to treat surgically induced pain has been dramatically restricted by the invasive nature of the available electrical leads that required a surgical incision to both insert and remove the multiple electrodes oriented in close proximity to the peripheral nerve [13]. In addition, the potential for nerve damage was not insignificant, and approximately one-quarter of reported implants resulted in lead migration or failure, necessitating surgical revision [10, 14–21]. Finally, fibrous capsule formation adherent to the target nerve frequently led to difficult lead removal [21]. Transcutaneous electrical nerve stimulation—involving the use of large skin electrodes—circumvents these limitations [22, 23]; but, skin pain fiber activation limits the amount of tolerated current, creating an unacceptably low “ceiling” effect [24].

To facilitate the application of peripheral nerve stimulation in treating pain resulting from surgical procedures such as total knee arthroplasty, an analgesic technique should optimally be administered without the requirement of an open surgical incision for either insertion or removal. This may be accomplished with the use of very small gauge electrical leads that allow the relatively rapid insertion via a percutaneously placed needle [25, 26]. Using ultrasound guidance to guide the insertion needle, a lead may be consistently introduced 0.5–3.0 cm from a peripheral nerve utilizing the same general landmarks and approach as for perineural nerve block administration [27, 28]. Ultrasound-guided percutaneous peripheral nerve stimulation was first reported in situ by Huntoon in 2009 using an epidural neurostimulation electrode for the treatment of chronic neuropathic pain [29]. Although related methods were later described for other chronic pain conditions [30–32], it has yet to be applied to a *post-surgical* pain state. We now report, to our knowledge, the first use of ultrasound-guided percutaneous peripheral nerve stimulation to treat post-surgical pain.

## Methods

### Consent to publish

This prospective feasibility study was conducted within the ethical guidelines outlined in the Declaration of Helsinki and followed Good Clinical Practice. Approval and oversight were provided by two Institutional Review Boards (Western IRB, Puyallup, WA; and Duke University Health System IRB, Durham, NC). An Investigational Device Exemption was granted for the use of these investigational devices by the US Food and Drug Administration (FDA), and written, informed consent was obtained from all subjects. The protocol was not registered as this was not a randomized, controlled trial.

We enrolled a convenience sample of adult subjects (21 years and older) with surgically related joint pain (≥3 on an 11-point numerical rating scale of the Brief Pain Inventory Short Form, Question 3: “Pain at its worst in the last 24 h”) uncontrolled with oral analgesics within 60 days following primary, unilateral, total (e.g., tricompartment) knee arthroplasty. Key exclusion criteria included the presence of implanted cardiac or deep brain stimulators, ongoing infections of the affected limb or other factors that increase the risk of infection, increased risk of bleeding (e.g., bleeding disorder), confounding pain conditions unrelated to the clinical indication for the knee arthroplasty (e.g., fibromyalgia), and nerve damage to the affected limb.

### Materials

**Fig. 1:**
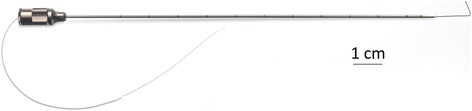
A 12.5 cm, 20 g needle with a pre-loaded helically coiled monopolar-insulated electrical lead (MicroLead, SPR Therapeutics, LLC, Cleveland, OH; illustration used with permission from Brian M. Ilfeld, MD, MS)

The investigational stimulation system used in the present study includes components (e.g., lead, stimulator) of a stimulation system that recently received FDA 510(k) clearance for the treatment of chronic and acute pain, including post-surgical and post-traumatic pain. Electrical stimulation was delivered through a helically coiled monopolar insulated electrical lead (MicroLead™, SPR Therapeutics, Cleveland, OH), which was pre-loaded in a 12.5 cm, 20 gauge introducer needle (Fig. 1). The lead was connected to a battery-powered electrical stimulator that was connected to the body via a surface return electrode (SPR Therapeutics, Cleveland, OH). To deliver test stimulation prior to lead placement, a 7.5 cm, 25 gauge or 12.5 cm, 24 gauge monopolar needle electrode (Test Needle, SPR Therapeutics, Cleveland, OH) was inserted. To guide placement of the lead and needle electrode, ultrasound imaging was used (M-Turbo, SonoSite, Bothell, WA; Flex Focus 400 exp, BK Medical, Peabody, MA) along with a linear array transducer (HFL38x 13–6 MHz 38 mm, SonoSite, Bothell, WA; Type 8870 18–6 MHz 60 mm, BK Medical, Peabody, MA) or curved array transducer (C60x 5–2 MHz 60 mm SonoSite, Bothell, WA; Type 8820e, 6–2 MHz 200 mm, BK Medical, Peabody, MA) to target femoral and sciatic nerves, respectively, within a sterile sleeve.

### Lead placement

**Fig. 2:**
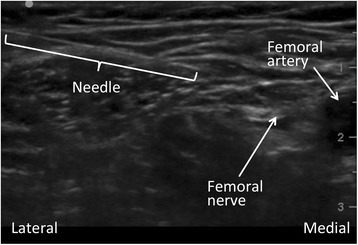
Ultrasound image of a femoral lead insertion

The anatomic lead location was determined by the origination of pain: anterior knee pain was treated with a femoral lead, and posterior knee pain received a sciatic lead. Subjects were positioned either supine or in the lateral decubitus position for femoral and sciatic insertions, respectively. Subjects had their ipsilateral limb prepared with chlorhexidine gluconate/isopropyl alcohol solution and sterile drapes at the level of the inguinal crease or over the gluteus maximus muscle for femoral and sciatic insertions, respectively. Nerves were imaged using ultrasound in a transverse cross-sectional (short axis) view at the inguinal crease for femoral leads and between the ischial tuberosity and greater trochanter for sciatic leads (Fig. 2). A local anesthetic skin wheal was raised lateral to the transducer, and no sedation was utilized.

To deliver test stimulation prior to lead placement, the monopolar needle electrode was inserted within the ultrasound plane and positioned approximately 0.5–1.0 cm from the femoral and 1.0–3.0 cm from the sciatic nerves. The electrical stimulator was used to deliver test stimulation (100 Hz, 15–200 μsec, 0.2–20 mA) to verify that comfortable sensations within the regions of pain could be induced without discomfort or evoking muscle contractions. If local cutaneous or subcutaneous discomfort was reported—indicating too superficial electrode placement—the needle electrode was advanced until resolution of the undesired sensations. If muscle contractions and/or discomfort distal to the lead insertion site were induced, the current intensity was reduced and/or the needle electrode was redirected until the contractions resolved.

**Fig. 3:**
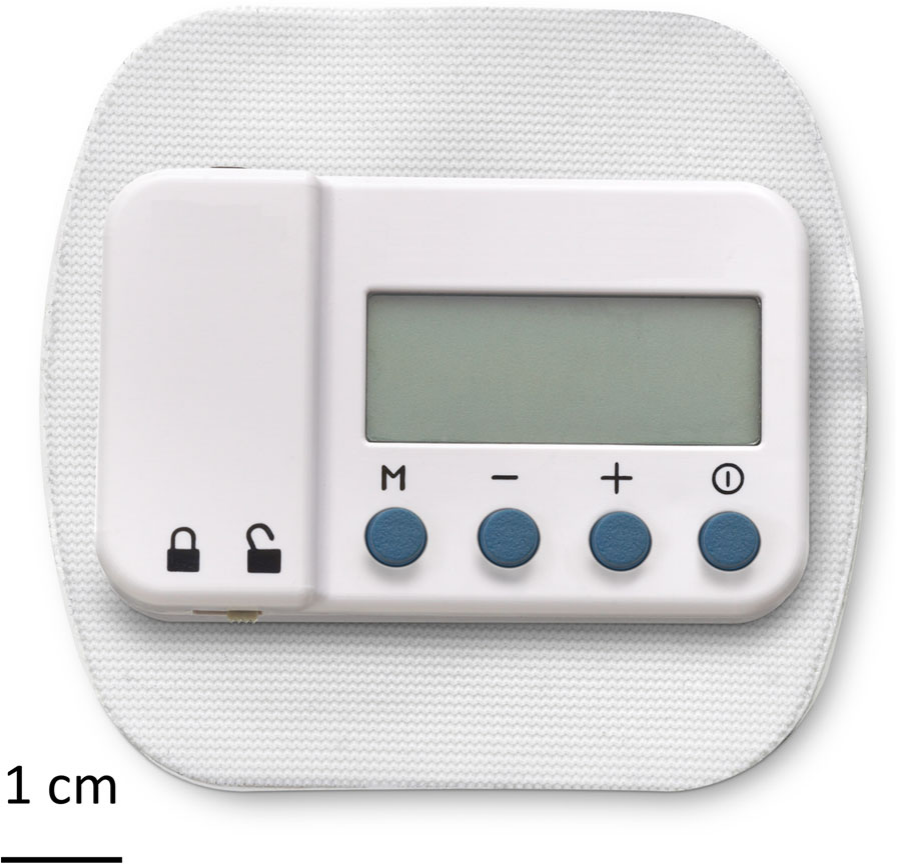
A stimulator attached to the surface return electrode (SPR Therapeutics, LLC, Cleveland, OH; figure used with permission from Brian M. Ilfeld, MD, MS)

Following a successful test (comfortable sensations and/or pain relief within the regions of pain without discomfort or muscle contractions), the monopolar needle electrode was withdrawn and replaced with the lead introducer needle (Fig. 1) using the same skin entry point and ultrasound approach. The final needle tip location was in the same location as the monopolar needle electrode (i.e., approximately 0.5–3.0 cm from the nerve). The needle was then withdrawn over the pre-loaded helically coiled electrical lead, the lead attached to a stimulator, and the stimulator subsequently attached to a surface return electrode (Fig. 3). Comfortable sensations over the regions of pain without muscle contractions confirmed accurate lead placement.

**Fig. 4:**
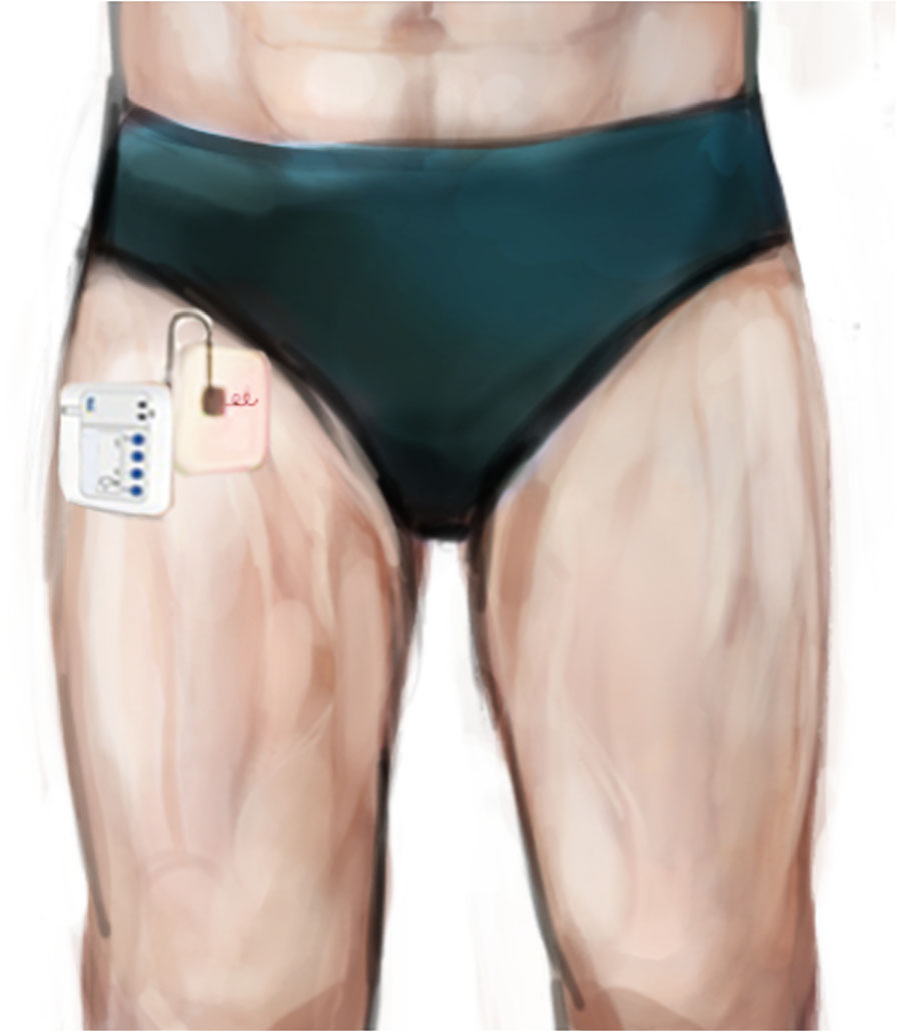
An electrical lead connected to a portable stimulator (SPR Therapeutics, LLC, Cleveland, OH; illustration used with permission from Brian M. Ilfeld, MD, MS)

A short portion of the lead outside the body was formed into a loop and affixed to the skin at the point of exit using an occlusive dressing (Fig. 4). For subjects with pain in the posterior aspect of the knee, a second lead was inserted between approximately 0.5–3.0 cm from the sciatic nerve between the greater trochanter and ischial tuberosity, using the technique described for the femoral lead. Following the measurement of the endpoints, the occlusive dressing was removed and gentle traction applied to the lead for extraction. A small sterile bandage was applied at the lead exit point.

### Outcome measures

Pain “right now” was evaluated first at rest and subsequently with passive and active knee motion using a Numeric Rating Scale of 0–10, with 0 and 10 equivalent to no pain and the worst imaginable pain, respectively (Question 6 of the Brief Pain Inventory, Short Form). Endpoints were evaluated immediately prior to lead insertion(s) and during the delivery of current. Passive and active knee range-of-motion was measured using a standard goniometer and included the number of degrees between the maximum flexion and extension measurements.

### Safety

Subjects received a telephone call 24 to 48 h after testing to evaluate the safety of the lead exit site. Also, subjects were instructed to contact the investigators for any device- or procedure-related adverse events after testing.

## Results

Five subjects were enrolled meeting all inclusion/exclusion criteria (Table 1). Leads were inserted without difficulty in all subjects; and stimulation produced comfortable sensations in the thigh/knee areas without discomfort or muscle contractions (Table 2). Percutaneous peripheral nerve stimulation produced immediate analgesia, decreasing pain an average of 93% at rest (mean NRS from 5.0 to 0.2) with 4 of 5 subjects experiencing complete resolution of pain (Table 3). In addition, pain during passive and active knee motion was reduced to 27 and 30%, respectively (Table 4). Neither maximum passive nor active knee range-of-motion was consistently affected (Table 5).

**Table 1:** Subject characteristics

Subject	Days since surgery	Diagnosis	Fixation method	Age (years)	Sex	BMI (kg/m^2^)	Leg
A	8	Osteoarthritis	Cement	48	Male	30	Left
B	9	Osteoarthritis	Cement	56	Female	26	Left
C	13	Osteoarthritis	Cement	73	Female	36	Left
D	41	Osteoarthritis	Unknown	66	Male	34	Left
E	58	Osteoarthritis	Unknown	61	Female	51	Left
Mean	26			61		35	

*BMI* body mass index

**Table 2:** Procedure-related pain and stimulation parameters

		Procedure-related pain	Stimulation parameters						
Subject	Catheter location	(NRS)	Minimum sensation		Maximum tolerable		Optimal settings		
			(μs)	(mA)	(μs)	(mA)	(μs)	(mA)	(Hz)
A	femoral	2	15	6	15	10	15	9	100
	*sciatic*		*18*	*20*	*18*	*20*	*18*	*20*	*100*
B	femoral	0	15	1	17	20	15	20	100
C	femoral	4	15	10	22	20	15	18	100
	*sciatic*		*15*	*20*	*200*	*20*	*50*	*20*	*100*
D	femoral	2	15	5	26	20	19	20	100
E	femoral	1	15	1	15	5	15	5	100
Mean		1.8	15	9	45	16	21	16	100

*NRS* numeric rating scale (0–10)

**Table 3:** Pain at baseline and during percutaneous peripheral nerve stimulation with electric current

Subject	Days since surgery	At rest		
		Stimulation		% Change
		Off	On	
A	8	3	1	67%
B	9	3	0	100%
C	13	7	0	100%
D	41	5	0	100%
E	58	7	0	100%
Mean	26	5.0	0.2	93%

Pain evaluated using a numeric rating scale (0–10)

**Table 4:** Pain at baseline and during percutaneous peripheral nerve stimulation with electric current

Subject	Days since surgery	During passive range-of-motion			During active range-of-motion		
		Stimulation		% Change	Stimulation		% Change
		Off	On		Off	On	
A	8	5	5	0%	5	5	0%
B	9	5	2	60%	6	4	33%
C	13	7	5	29%	6	4	33%
D	41	9	8	11%	9	6	33%
E	58	6	3	50%	6	4	33%
Mean	26	6.4	4.6	30%	6.4	4.6	27%

Pain evaluated using a numeric rating scale (0–10)

**Table 5:** Range-of-motion at baseline and during percutaneous peripheral nerve stimulation with electric current

	Passive range-of-motion			Active range-of-motion		
	Stimulation		Change	Stimulation		Change
Subject	Off	On		Off	On	
A	61	60	−1	57	53	−4
B	21	39	18	10	54	44
C	63	70	7	75	61	−14
D	111	116	5	100	110	10
E	88	80	−8	87	88	1
Mean	69	73	4	66	73	7

Active and passive knee range-of-motion was measured using a standard goniometer and included the number of degrees between the maximum flexion and extension measurements

All leads were removed without difficulty approximately 1–2 h after the start of testing. There were no device-related adverse events.

## Discussion

This prospective feasibility case series suggests that ultrasound-guided percutaneous peripheral nerve stimulation may be applicable to pain following total knee arthroplasty. The relatively recent convergence of five advances may now allow the wide application of this modality to treat post-surgical pain: (1) the recent propagation of ultrasound machines available to practitioners for use in regional analgesia; (2) the current pervasiveness of anesthesiologists trained in ultrasound-guided regional anesthesia; (3) an insulated electrical lead specially developed for percutaneous insertion and extended use adjacent to peripheral nerves that enables selective activation of pain-relieving fibers when inserted remote (approximately 0.5–3.0 cm) from the target nerve; (4) a novel stimulator that may be adhered directly to the skin due to its small footprint and slim design; and (5) the recent FDA 510(k) clearance of this percutaneous peripheral nerve stimulation system for the treatment of chronic pain and acute pain, including post-surgical and post-traumatic pain.

**Fig. 5:**
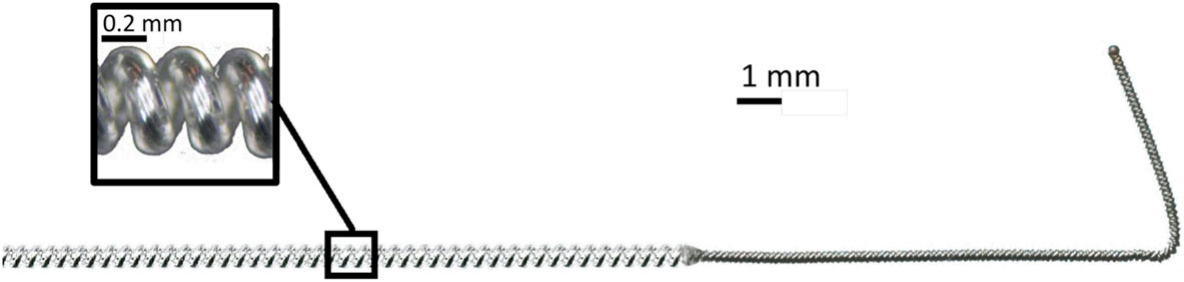
A small-diameter (0.2 mm) open-coiled, helical electrical lead with an anchoring wire (MicroLead, SPR Therapeutics, LLC, Cleveland, OH; figure used with permission from Brian M. Ilfeld, MD, MS)

The novel electrical leads used in this investigation were comprised of a small-diameter open helical coil (0.2 mm wire diameter and 0.6 mm overall coil diameter) wound from a fluoropolymer insulated 7-strand, type 316L stainless steel wire with a single terminal anchor at the tip (Fig. 5). The lead was specifically designed to provide multiple advantages that increase the applicability of nerve stimulation to the management of post-surgical pain. Percutaneous insertion with a 20 g needle is possible due to the relatively small coil diameter (Fig. 1), and removal may be achieved simply with continuous traction. The helical shape theoretically decreases the incidence of fracture and migration, as well as lowering the risk of infection to less than 0.1% for up to 60 days [33]. The minimal infection risk and investigational device exemption (IDE) from the US FDA for use up to 60 days in clinical investigations raises the possibility of providing post-surgical analgesia that outlasts the pain resulting from not only total knee arthroplasty but the overwhelming majority of orthopedic procedures. It is for this reason that we included subjects who were within 60 days of surgery for the current investigation.

**Fig. 6:**
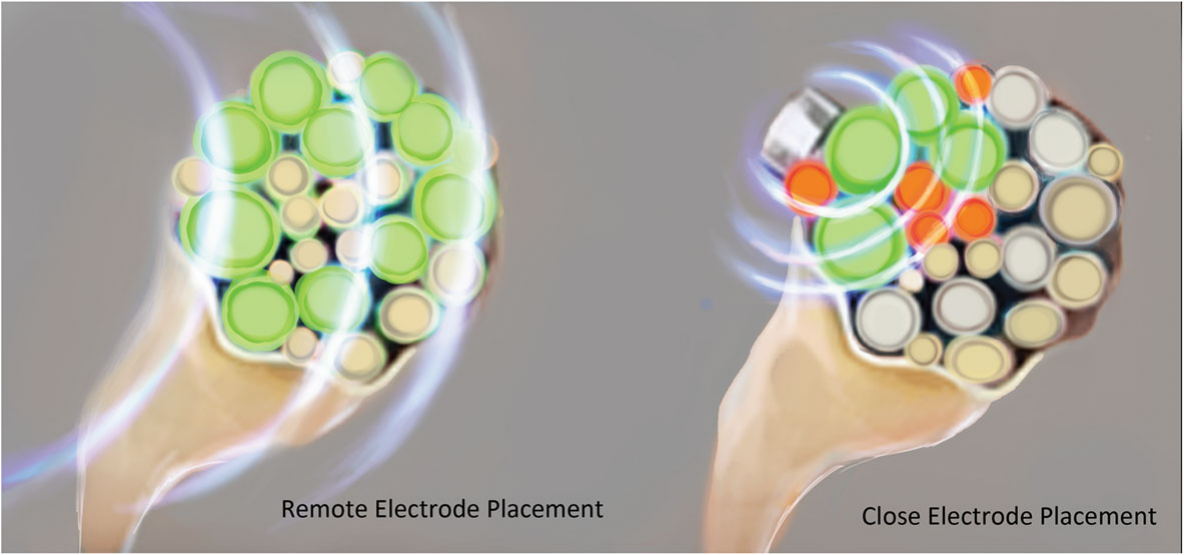
The therapeutic window and the ability to preferentially activate the targeted large nerve fibers—without activating non-targeted pain or motor neurons—increases as the distance between the electrode and the nerve increases (illustration used with permission from Brian M. Ilfeld, MD, MS)

The single coiled monopolar lead enables a stimulation paradigm that is intended to provide pain relief while minimizing muscle contractions and discomfort from stimulation. The challenge with peripheral nerve stimulation for the treatment of pain has been to activate selectively the pain-relieving (large-diameter myelinated) fibers within a nerve trunk while avoiding activation of smaller diameter (alpha motoneurons, or types III and IV) fibers. This “selective activation” of large over small diameter fibers improves as pulse duration decreases [34] and the distance between the electrode and nerve increases [35]. Compared to conventional peripheral nerve stimulation that uses multi-electrode leads placed close to the nerve (commonly ≤0.2 cm) and wide pulse durations (90–500 μs), the stimulation paradigm in the present study utilized remote lead placement (approximately 0.5–3.0 cm from the nerve) and narrow pulse durations (15–50 μs). Such remote lead placement is enabled by the ability of the single, coiled monopolar lead to resist migration due to its coiled structure and terminal anchor at the electrode (Fig. 6).

In addition, the positive results of the present study are unlikely to be due to peripheral nerve field stimulation, where electrodes are placed subcutaneously near the regions of pain to activate adjacent nerve fibers to generate sensations locally to relieve pain. In contrast, the present technique directly stimulated peripheral nerves proximal to the surgical wound to produce sensations and analgesia distal to the leads within the nerve distributions.

Percutaneous peripheral nerve stimulation produced immediate reductions in pain that compare favorably to existing treatments for postoperative pain. Pain at rest was completely relieved in 4 of 5 subjects (overall average relief = 93%), and pain during passive and active flexion was decreased 27 and 30%, respectively. Although one subject did not achieve complete relief of pain at rest with stimulation on (pain = 1) compared to stimulation off (pain = 3), adjustment of the lead location and/or stimulation parameters may have enabled this subject to achieve complete pain relief. With this small feasibility study, we can only speculate on the degree of pain control provided with percutaneous peripheral nerve stimulation relative to single injection and continuous peripheral nerve blocks [4]. However, it is notable that adductor canal blocks reduced pain to a similar degree following total knee arthroplasty, albeit immediately following surgery [36, 37].

Ultrasound-guided percutaneous peripheral nerve stimulation has several important limitations. Small lead fragments, typically less than 0.1 mm^3^ in volume (less than a tenth of the volume of a common skin staple) and 0.8 mg in weight, may be retained upon withdrawal of the lead at the end of the therapy period. MRI scanning may still be performed on a patient with a retained fragment since the retained fragments are MR conditional using common scanning conditions at up to 1.5 Tesla (standard field strength used clinically) [38]. Also, no lead fractures have been reported within the body during therapy and retained fragments have not produced complications when left in situ and occur in less than 8% of cases (20 of 267) when used for the treatment of pain [24, 30, 31, 39–46].

Furthermore, the subjects of this feasibility study underwent treatment 8–58 days following surgery, and the quality of pain control and impact on supplemental analgesic consumption provided within the first postoperative week remains unknown. Lastly, the subjects of this pilot study underwent stimulation for less than 1 h, while the desirable duration of treatment following total knee arthroplasty would be far longer.

How practical ultrasound-guided percutaneous peripheral nerve stimulation is following total knee arthroplasty as an alternative to opiates and other analgesic techniques will be determined with further research. Ongoing studies are underway with the goal of evaluating safety (e.g., ability to reduce risks of falls relative to existing therapies), efficacy (including during the first postoperative week as well as after the end of stimulation therapy), the potential placebo effect, and the value of the therapy relative to its costs. However, the data provided by the current feasibility study suggest that there is immense potential for making a historic advancement in the treatment of post-surgical pain.

## Conclusions

This prospective feasibility case series suggests that ultrasound-guided percutaneous peripheral nerve stimulation may be applicable to pain following total knee arthroplasty and possibly other orthopedic surgical procedures. If subsequent studies demonstrate a favorable risk-benefit ratio, *the modality has the possibility to entirely transform post-surgical pain control as it has been performed administering local anesthetic for over 100 years* [47].

## Abbreviations

cm: Centimeters
Fig: Figure
g: Gauge
Hz: Hertz
mA: Milliamps
mm: Millimeters
NRS: Numeric rating scale
™: Trademark
μsec: Microsections
